# Stool and vaginal microbiome profiles patterns among Black and White endometrial cancer survivors: A pilot study in North Carolina

**DOI:** 10.1371/journal.pone.0336772

**Published:** 2026-01-23

**Authors:** Mu Jin, Temitope Keku, Amber McCoy, Jordyn A. Brown, Marc Peterson, Jamie Hunter, Shawn Smith, Stephenie Black-Grant, Melinda S. Yates, Anne F. Peery, Victoria L. Bae-Jump, M. Andrea Azcarate-Peril, Stephanie M. Engel, Andrew F. Olshan, Hazel B. Nichols

**Affiliations:** 1 Department of Epidemiology, University of North Carolina (UNC) Gillings School of Global Public Health, Chapel Hill, North Carolina, United States of America; 2 Department of Medicine, Division of Gastroenterology and Hepatology, Center for Gastrointestinal Biology and Disease, UNC School of Medicine, Chapel Hill, North Carolina, United States of America; 3 UNC Lineberger Comprehensive Cancer Center, Chapel Hill, North Carolina, United States of America; 4 Endometrial Cancer Action Network for African Americans, Seattle, Washington, United States of America; 5 Department of Pathology & Laboratory Medicine, UNC School of Medicine, Chapel Hill, North Carolina, United States of America; 6 Division of Gynecologic Oncology, University of North Carolina, Chapel Hill, North Carolina, United States of America; 7 UNC Microbiome Core, University of North Carolina, Chapel Hill, North Carolina, United States of America; Washington State University - Spokane, UNITED STATES OF AMERICA

## Abstract

**Background:**

Endometrial cancer is the most common gynecologic cancer in the US. Endometrial cancer survivors may experience changes in microbiome due to cancer treatment and other factors. The human microbiome plays a crucial role in maintaining the proper functioning of the body. A more diverse microbiome often indicates a healthier gut environment, while lower vaginal microbiome diversity, specifically a *Lactobacillus*-dominant vagitype, is associated with more favorable health outcomes. The objectives of this pilot study were to evaluate potential variation in stool and vaginal microbiome communities and to assess the feasibility and acceptability of self-sampling among endometrial cancer survivors.

**Methods:**

Endometrial cancer survivors (N = 50) enrolled in the Carolina Endometrial Cancer Study, a cohort of women diagnosed with endometrial cancer, were mailed Genotek vaginal swab and stool self-collection kits. Self-reported questionnaires assessed information on survivors’ demographics, sexual and bowel function, and perspectives on the self-sampling processes. Tumor characteristics and cancer treatment information were assessed from medical records. Microbiota profiles were characterized by bacteria 16S rRNA amplicon sequencing.

**Results:**

Overall, 48 vaginal swabs and 47 stool samples were obtained. Alpha (Shannon *p* = 0.04) and beta (Bray-Curtis *p* = 0.004) diversity of vaginal microbiome samples varied by cancer treatment, with higher microbial diversity after chemotherapy or radiation compared to surgery alone. In the surgery only group, 63% of samples were *Lactobacillus*-dominant compared to 17% among the chemotherapy or radiation group. Stool microbiome diversity did not vary by cancer treatment status. No statistically significant differences in alpha or beta diversity were observed in either vaginal or stool microbiome communities across racial subgroups or by sexual or bowel function.

**Conclusion:**

Self-collection of stool and vaginal microbiome samples is feasible and acceptable in cancer survivors. Our results suggest that radiation and chemotherapy for endometrial cancer may decrease the abundance of beneficial *Lactobacillus* and increase less favorable vaginal microbial diversity among endometrial cancer survivors.

## Introduction

In the United States, endometrial cancer survivors are the second largest group of female cancer survivors (after breast), with >890,000 women living with an endometrial cancer history [1]. In recent decades, endometrial cancer incidence and mortality have increased with stark racial disparities between Black and White women [2,3]. Black women are more often diagnosed at more advanced stages with tumors that have poor prognosis features, such as non-endometrioid histology, requiring more intensive cancer therapies [4–6]. Even when accounting for histologic stage, Black women are more likely to have their cancer recur and our twice as likely to die compared to their White counterparts [7].

The human microbiome is the collection of microorganisms that live together in the human body [8] and comprises bacteria, archaea, fungi, algae, and small protists [9,10]. The microbiome plays a vital role in numerous processes, including facilitating nutrient absorption [11], protecting against harmful pathogens [12], and supporting the development of the immune system [13]. Microbiome dysbiosis, an imbalance in the composition of the microbiome, is increasingly recognized for its potential role in cancer development and treatment response [14–16]. Cancer treatments such as chemotherapy and radiation can induce stool and vaginal microbiome dysbiosis, which may contribute to treatment-related toxicities, thus impact patients’ health outcomes [17–20].

In general, a healthy gut microbiome community is characterized by high taxonomic diversity, while lower diversity can be a sign of dysbiosis [21–23]. Lower diversity of the gut microbiome has been associated with several acute and chronic diseases, such as obesity, inflammatory bowel diseases and colorectal cancer [24,25]. Unlike the beneficial high diversity that characterizes the gut microbiome, a healthy vaginal microbiome is frequently characterized by low diversity dominated by *Lactobacillus* species [26]. This contrast in the relative benefit of high diversity between gut and vaginal profiles may be partially explained by the observation that a *Lactobacillus-*dominated vaginal microbiome contributes to a lower vaginal pH, which may be beneficial for women’s health by preventing infections or vaginal inflammation [27–32]. Several studies have observed that *Lactobacillus-*dominated vaginal microbiome profiles are associated with a lower probability of experiencing adverse health conditions such as bacterial vaginosis, sexually transmitted infections, and adverse reproductive outcomes [33–37].

Cancer treatment-related changes in microbiome composition have the potential to contribute to endometrial cancer outcomes during survivorship, including patient-reported concerns about bowel and sexual function [38,39] and racial disparities in recurrence risk [40,41]. In several non-cancer studies, Black women were less likely to have *Lactobacillus-*dominated vaginal microbiome profiles [40–44] and had lower gut microbiome diversity [45,46] compared to White women.

The objective of this pilot research was to explore potential variation in stool and vaginal microbiome communities by race and cancer treatment. We also evaluated whether stool microbial composition was related to patient-reported bowel function and whether vaginal microbial composition was related to patient-reported sexual function. We further assessed the feasibility and acceptability of stool and vaginal self-sampling among racially diverse endometrial cancer survivors.

## Methods

The Carolina Endometrial Cancer Study (CECS) is an ongoing population-based cohort of endometrial cancer survivors in North Carolina; participant enrollment began in 2021. Women with recently diagnosed endometrial cancer are identified through a Rapid Case Ascertainment collaboration between the North Carolina Central Cancer Registry and the University of North Carolina (UNC) Lineberger Comprehensive Cancer Center. In the full cohort, all study activities are conducted remotely. Participants complete surveys online, on the phone, or by mail and provide a saliva specimen through the mail for germline DNA collection. Medical records and tumor specimens are requested from the treating hospital with participant consent. All study activities are approved by the Institutional Review Board of the University of North Carolina (#19–1928 and 23–0666).

### Home visit pilot study

From December 10, 2021, to July 21, 2022, we conducted nurse home visits for a pilot study of 50 CECS participants who lived within a 100-mile (or 2-hour travel) radius of UNC Chapel Hill, as previously described [47]. In brief, at enrollment into the parent CECS cohort, participants were asked about their willingness to participate in a home visit and provide self-collected vaginal swab and stool samples to assess the potential for future mail-based collection of microbiome self-sampling kits. The written informed consent was sent to participants via mail and was then collected by nurses during home visits. In advance of the nurse visit, participants were mailed questionnaires and Genotek vaginal and stool self-collection kits (vaginal swab, fecal spatula, and stabilization solutions). For vaginal swab self-collection, cancer survivors were asked to wash their hands, sit comfortably, and avoid collection during menstruation. They were instructed to insert the swab 3–5 cm into the vagina, rotate it in full circles for 20 seconds, and then carefully remove it. The swab was then placed into a collection tube with stabilizing liquid, snapped at the break point, and sealed tightly. For the stool sample, participants were instructed to use the kit-provided spatula to transfer a small amount of fecal matter into a collection tube containing stabilizing liquid. They then leveled off excess sample, securely sealed the tube, and shook it for 30 seconds to mix with the stabilizing liquid. The participants were asked to store the collection tube at room temperature (15–25 °C).

During the scheduled home visit, nurses retrieved surveys and kits, measured height, weight, waist and leg circumferences, and reviewed medications. The average time between specimen collection and nurse retrieval was < 2 days. The samples were then delivered, stored, and analyzed at the UNC Microbiome Core. The final pilot sample consisted of 27 White and 23 Black participants.

### DNA extraction and sequencing

Stool and vaginal microbiome samples were analyzed by 16S rRNA high throughput sequencing at the UNC Microbiome Core. Genomic DNA was extracted using a modified protocol of the Qiagen Dneasy Kit (Qiagen, Germantown, MD). The bacterial 16S rRNA sequences were filtered to remove low quality reads and processed through QIIME 2. Sequences were assorted to operational taxonomic units using the Silva Database. Six control samples (four negative controls and two positive controls (Zymo D6300) [48]) were considered for de-contamination; taxa likely to be contaminants were excluded from the analysis.

To minimize host-derived sequence contamination in the vaginal microbiome data, all reads that could not be taxonomically assigned at the genus level were excluded, leaving 25 bacterial genera for downstream analysis. For the gut microbiome, we selected 24 genera *a priori* based on their reported associations with gynecologic malignancies in the literature [49] to focus our analyses on taxa most likely to be biologically relevant.

### Survey measures

All CECS study participants completed interviews by phone, via an online portal, or by mailed questionnaires that included self-reported race. The Home Visit Pilot Study sample additionally completed mailed questionnaires that included questions on sexual and bowel function scales, use of antibiotics in the prior month, other prescription medications, non- prescription medications/vitamins/supplements, yeast infections or sexually transmitted infections in the past two months, and whether they had ever experienced bacterial vaginosis. Sexual function was assessed by the validated Sexual function-Vaginal changes Questionnaire (SVQ), which is designed to evaluate intimacy, sexual satisfaction, sexual interest, vaginal changes, and sexual functioning among gynecologic cancer survivors [50]. The SVQ includes a total of 25 items and is comprised of three parts. Part 1 includes 11 items that span intimacy (close physical contact), sexual relations, worry about sex, partner status, and interest and satisfaction with sex and appearance during the last month. Part 2 is completed only by participants who have been sexually active in the last month and includes 7 items that query vaginal dryness, pain, bleeding, vaginal size, completion, orgasm, and relaxation. Part 3 focuses on changes since a cancer diagnosis and includes 3 items asked of all participants (whether interest in close physical contact or sexual relations has changed); 1 question asked of those with a partner (changes in partner’s interest in sexual relations); and 3 questions asked of those who are sexually active (changes in dryness, vaginal size, and pain).

The validated Colorectal-Anal Distress Inventory 8 (CRADI-8) questionnaire, a subscale of the Pelvic Floor Disability Index (PFDI-20), assessed the degree of bother and distress caused by bowel symptoms, including straining with bowel movements, sensation of incomplete bowel movements, pain with defecation, fecal urgency, and fecal incontinence [51].

In addition, participants shared their experiences with stool and vaginal swab collection. Among the participants who submitted a sample, they indicated their level of comfort on a 5-point Likert scale ranging from “very comfortable” to “very uncomfortable.” Respondents who refused to provide a sample were asked why a sample was not collected and reason(s) why they chose not to provide a sample. All participants were asked to qualitatively describe why they felt uncomfortable providing a sample or provide suggestions to improve the collection process.

### Medical record abstraction

With participant consent, medical records and tumor specimens were requested from hospitals statewide across North Carolina. Medical record abstraction included, but is not limited to, tumor stage, histology, and cancer treatments received, including the surgical approach, adjuvant radiotherapy modality (brachytherapy, external beam, etc.) and chemotherapy (regimen, agents, dose, and number of cycles). For this analysis, tumor characteristics and cancer treatment receipt and recency were abstracted from medical records. A final coded dataset (without individual identifying information) for this analysis was provided on February 17, 2025.

### Analysis and statistical approach

The SVQ includes domain scores for intimacy (possible range = 2–8), sexual interest (possible range 1–4), global sexual satisfaction (possible range = 2–11), satisfaction with appearance (possible range 1–7), and changes in intimacy and sexual interest (possible range 3–9). Higher SVQ scores indicate worse sexual function. We categorized SVQ domain scores at <versus ≥ the median value in our sample: intimacy (<5 and ≥5), sexual interest (<2 and ≥2), global sexual satisfaction (<7 and ≥7), satisfaction with appearance (<5 and ≥5), and changes in intimacy and sexual interest (<6 and ≥6).

The CRADI-8 includes 8 questions focused on the presence and severity of bowel symptoms over the last three months. The scale includes a binary response (“No” or “Yes”) for symptom presence and, if “Yes,” a four-point Likert scale (“Not at all” to “Quite a bit”) for bother severity. Each question was scored using both the binary response and the four-point Likert-type scale on a 0–4 scale, with 0 representing “No”, 1 representing “Not at all”, and 4 representing “Very much”. The 0–4 score for each of the 8 items were summed for a possible range of 0–32. Higher CRADI-8 scores indicate worse bowel function. In our sample, CRADI-8 scores ranged from 0–19 and were categorized in tertiles (≤ 4, 5-7, and ≥ 8).

For alpha diversity analysis, we computed the Chao1 index (a measure of species richness) [52] and the Shannon diversity index (a measure of species richness and evenness) [53]. Higher alpha diversity in gut microbiome usually indicates better health status of gut [21–23], though lower alpha diversity is more beneficial in the vaginal microbiome. Beta-diversity was defined by the Bray-Curtis distance [54]. *P*-values for group differences in alpha and beta diversity were calculated using Mann–Whitney-U tests and PERMANOVA, respectively [55]. For vaginal microbiome samples, we additionally classified vagitypes as *Lactobacillus*-dominant or non-dominant based on the relative abundance of *Lactobacillus* (>50% vs. ≤ 50%) [56,57]. *P*-values <0.05 were used as the threshold for statistical significance. We performed differential abundance analysis using edgeR at the genus level to compare microbial composition across treatment and histology type subgroups. Log_2_ fold change (log_2_FC) values were calculated to quantify differences in genus abundance between groups, and statistical significance was assessed using p-values, with multiple comparisons corrected via the Benjamini-Hochberg False Discovery Rate (FDR). Genera with an FDR < 0.05 were considered statistically significant. MicrobiomeAnalyst 2.0 [58] and R 4.3.2 [59] were used for analysis. Cell sizes <5 are suppressed to protect patient confidentiality in accordance with requirements from the North Carolina Central Cancer Registry.

## Results

### Study population

Among 50 participants who completed home visits, 27 were White and 23 were Black. On average, participants were 61.3 years old (SD = 10.1 years) at enrollment and 16.5 months (SD = 5.3) from endometrial cancer diagnosis. Among 48 participants with cancer treatment information, 25 (52.1%) had surgery only and 23 (47.9%) had either radiation or chemotherapy. The majority of participants had grade 1 or 2 tumors (n = 33, 70.2%), while 27.7% had grade 3 tumors (n = 13). Most tumors were classified as endometrioid (n = 37, 74%), 26% (n = 13) were non-endometrioid (**Table 1**). Participant characteristics among women who provided a stool or vaginal sample are also provided in **Table 1**.

**Table 1:** Characteristics of the Carolina Endometrial Cancer Study participants who participated in the home visit pilot study with vaginal swab and stool sample self-collection.

Participant characteristics	Overall (N = 50)	Vaginal microbiome (N = 40^a^)	Stool microbiome (N = 39^b^)
**Age at home visit (years)**; Mean (SD)	61.3 (10.1)	62.0 (9.1)	61.5 (8.7)
**Age at home visit (years)**			
**≤ 65**	30 (60.0)	23 (57.5)	23 (59.0)
**> 65**	20 (40.0)	17 (42.5)	16 (41.0)
**Months from diagnosis to home visit**; Mean (SD)	16.5 (5.3)	16.9 (5.0)	16.9 (5.0)
**Race**			
White	27 (54.0)	20 (50.0)	19 (48.7)
Black	23 (46.0)	20 (50.0)	20 (51.3)
**Body mass index** (BMI, kg/m^2^); Mean (SD)	34.8 (8.1)	34.7 (7.7)	34.9 (7.7)
**Obesity classes**			
Not obese (BMI < 30)	16 (32.0)	12 (30.0)	11 (28.2)
Class I (30 ≤ BMI < 35)	9 (18.0)	9 (22.5)	9 (23.1)
Class II (35 ≤ BMI < 40)	11 (22.0)	9 (22.5)	9 (23.1)
Class III (BMI ≥ 40)	14 (28.0)	10 (25.0)	10 (25.6)
**Cancer treatment modalities**			
Surgery only^c^	25 (52.1)	19 (48.7)	19 (50.0)
Any radiation or chemotherapy	23 (47.9)	20 (51.3)	19 (50.0)
**Grade**			
Grade 1 or 2	33 (70.2)	28 (71.8)	27 (71.1)
Grade 3	13 (27.7)	11 (28.2)	11 (28.9)
**Histology**			
Endometrioid	37 (74.0)	29 (72.5)	28 (71.8)
Non-endometrioid	13 (26.0)	11 (27.5)	11 (28.2)
**Bacterial vaginosis (ever)**			
No	39 (83.0)	32 (82.1)	31 (81.6)
Yes	8 (17.0)	7 (17.9)	7 (18.4)
**Vaginal yeast infection (within 2 months)**			
No	44 (93.6)	36 (92.3)	35 (92.1)
Yes	<5^d^	<5	<5
Missing	<5	<5	<5
**Sexually transmitted infection (within 2 months)**			
No	47 (94.0)	39 (97.5)	38 (97.4)
Yes	<5	<5	<5
Missing	<5	<5	<5

^a^Two did not provide vaginal swabs, eight excluded due to antibiotic use in the last month

^b^Three did not provide stool samples, eight excluded due to antibiotics use in the last month

^c^Includes one participant who had received no treatment at the time of sampling

^d^Cells with sizes <5 are suppressed to protect patient confidentiality in accordance with requirements from the North Carolina Central Cancer Registry.

### Feasibility of stool sampling

**Fig 1:**
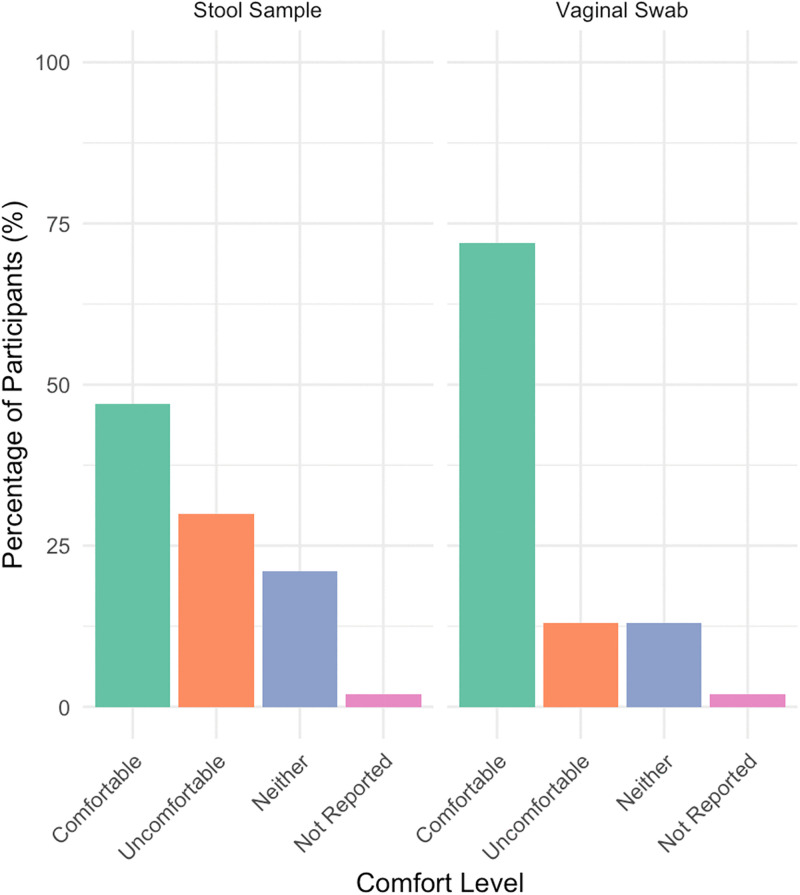
Distribution of self-reported comfort levels with the collection process for stool (N = 43) and vaginal swab (N = 47) samples.

A total of 47 stool samples were successfully collected. At the time of the home visit, 43 participants submitted a stool sample with the remaining four participants submitting a sample by mail after the home visit. Among the 43 participants who submitted a stool sample at the time of the home visit, 47% were comfortable, 21% were neither comfortable nor uncomfortable, 30% were uncomfortable, and 2% did not report their level of comfort with providing a stool sample (**Fig 1**). Participants who volunteered feedback on the stool sampling process described the process as “disgusting,” and found the stool collection kits challenging and inconvenient, recommending more durable materials in future kits.

### Participant characteristics and stool microbial composition

**Fig 2:**
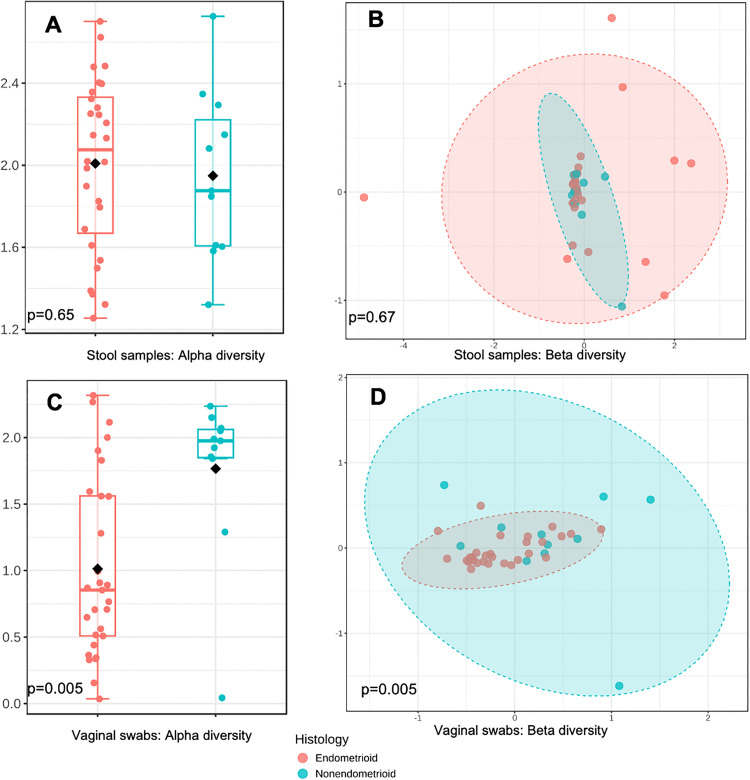
Stool sample and vaginal swab microbiome diversity profiles according to histology type (endometrioid vs. non-endometrioid): a. alpha diversity (Shannon index) for stool sample microbiome; b. beta diversity (Bray-Curtis) for stool sample microbiome; c. alpha diversity (Shannon index) for vaginal swab microbiome; d. beta diversity (Bray-Curtis) for vaginal swab microbiome.

**Fig 3:**
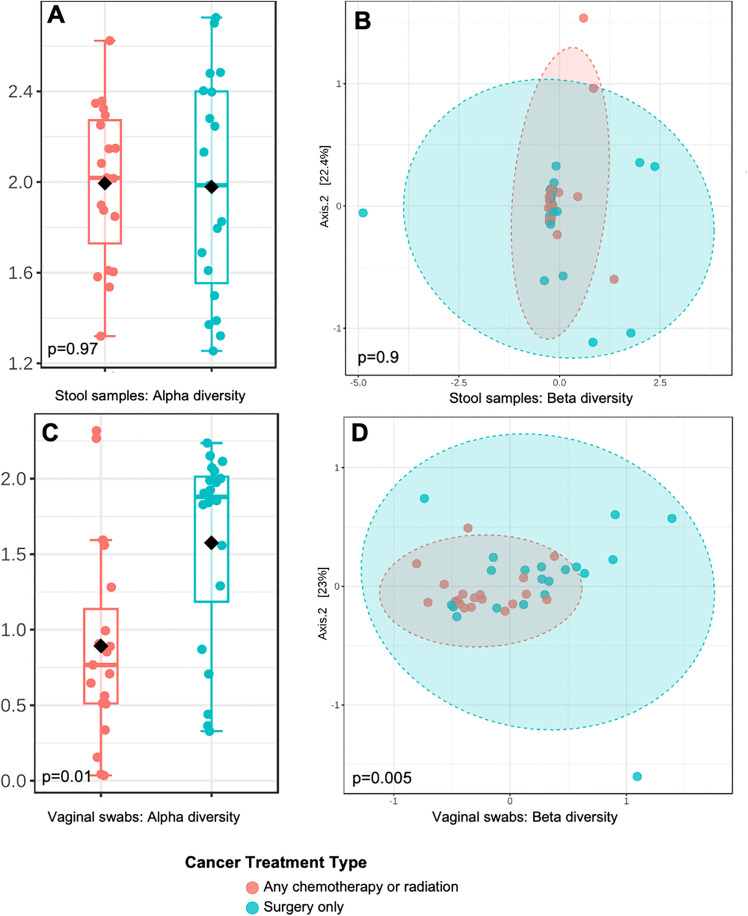
Stool sample and vaginal swab microbiome diversity profiles according to cancer treatment (any chemotherapy/radiation vs. surgery only): a. alpha diversity (Shannon index) for stool sample microbiome; b. beta diversity (Bray-Curtis) for stool sample microbiome; c. alpha diversity (Shannon index) for vaginal swab microbiome; d. beta diversity (Bray-Curtis) for vaginal swab microbiome.

From the 47 participants with a stool sample, we excluded eight participants who reported antibiotic use in the month prior to sample collection, resulting in 39 samples available for analysis. Among these 39, the most abundant species were *Bacteroides*, *Prevotella*, and *Blautia*. Neither race (Shannon p = 0.9, Chao1 p = 0.3, Bray-Curtis p = 0.9,), cancer treatment (Shannon p = 0.7, Chao1 p = 0.6, Bray-Curtis p = 0.8, **Fig 2**), nor cancer histology (Shannon p = 0.7, Chao1 p = 0.5, Bray-Curtis p = 0.7, **Fig 3**) were associated with significant differences in alpha or beta diversity in the stool samples.

### Stool microbial composition and patient-reported bowel symptoms

Among the 39 participants who submitted evaluable stool samples, the mean CRADI-8 score was 6.33 (SD = 5.2), with a median score of 5 and tertile cutoffs at 3.7 and 7.3. We assessed alpha and beta diversity of stool microbiome samples according to tertiles of the CRADI-8 score. Stool microbiome alpha (Shannon p = 0.8, Chao1 = 0.4) and beta (Bray-Curtis p = 0.5) diversity scores did not differ significantly between the highest tertile and lowest tertile of CRADI-8 scores (**S1 Fig**).

### Feasibility of vaginal swab collection

Of the 48 participants who submitted a vaginal swab sample at the time of the home visit, the majority (72%) reported they were comfortable with the process (**Fig 1**). Few participants did not provide samples due to disability or having replacement kit in transit (but ultimately did not return the kit). Participants who provided additional feedback on the vaginal self-sampling process volunteered that they perceived the process to be difficult as it required the use of a hand mirror, had uncertainty about the insertion distance in the vaginal canal, and reported side effects such as vaginal bleeding and cramping.

### Participant characteristics and vaginal microbial composition

**Fig 4:**
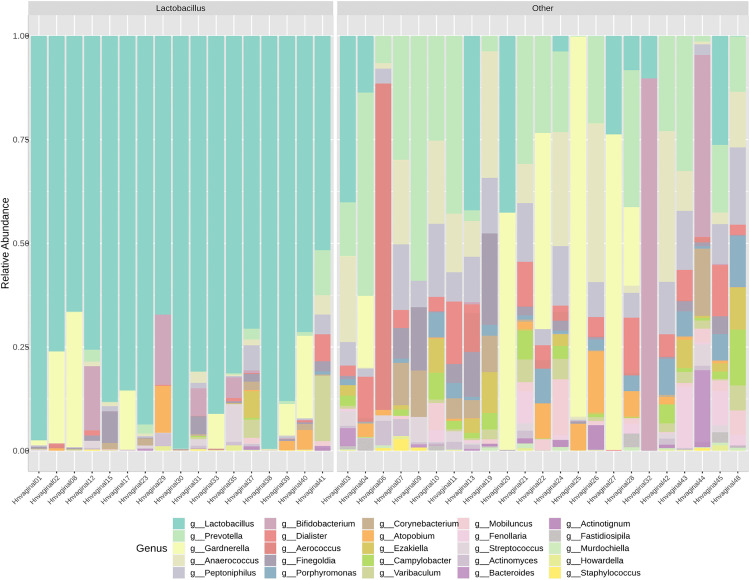
Relative abundance of vaginal microbiome genera by vagitype (*Lactobacillus* dominant vs. non-*Lactobacillus* dominant vagitypes).

We excluded 8 samples from participants who used antibiotics one month before sample collection, resulting in 40 vaginal samples available for analysis. *Lactobacillus*, *Gardnerella*, and *Prevotella* were the most abundant genera. We clustered vaginal samples into two subgroups: *Lactobacillus*-dominant (n = 17) and the non-*Lactobacillus*-dominant (n = 23) based on the relative abundance of *Lactobacillus* (**Fig 4**). Race was not associated with vaginal alpha diversity (Shannon p = 0.7, Chao1 p = 0.5), beta diversity (Bray-Curtis p = 0.1), or vagitype (p = 0.3). Although not statistically different (p > 0.05), 50% (n = 10) of White participants had *Lactobacillus*-dominant vaginal microbiome profiles compared to 35% (n = 7) of Black participants (**Table 2**).

**Table 2:** Characteristics of the vaginal microbiome study samples by vagitype (N = 40).

Participant characteristics	*Lactobacillus*-dominant	Non-*Lactobacillus*-dominant	p-value
**Total**	17	23	
**Age at home visit (years)**; Mean (SD)	59.6 (11.9)	63.8 (6.1)	0.5
**Age at home visit (years)**			0.9
**≤ 65**	10 (58.8)	13 (56.5)	
**> 65**	7 (41.2)	10 (43.5)	
**Body mass index** (BMI, kg/m^2^); Mean (SD)	34.0 (7.0)	35.2 (8.3)	0.6
**Obesity classes**			0.4
Not obese (BMI < 30)	5 (29.4)	7 (30.4)	
30 ≤ BMI < 37	7 (41.2)	5 (21.7)	
BMI ≥ 37	5 (29.4)	11 (47.8)	
**Race**			
White	58.8%	43.5%	0.3
Black	41.2%	56.5%	
**Cancer treatment modalities**			<0.001
Surgery only^a^	81.3	26.1	
Any radiation or chemotherapy	18.8	73.9	
**Grade**			0.5
Grade 1 or 2	81.3%	65.2%	
Grade 3	18.8%	34.8%	
**Histology**			0.01
Endometrioid	94.1%	56.5%	
Non-endometrioid	5.9%	43.5%	
**Sexually active**			0.3
No	52.9%	69.6%	
Yes	47.1%	30.4%	
**Global Sexual Satisfaction**			
Mean score	7.2 (3.0)	9.0 (2.1)	
< 7	58.8%	36.4%	0.2
≥ 7	41.2%	63.6%	
**Intimacy**			
Mean score	5.4 (1.9)	5.2 (1.9)	
< 5	29.4%	45.5%	0.3
≥ 5	70.6%	54.5%	
**Sexual Interest**			0.8
No Sexual interest	35.3%	39.1%	
Had sexual interest	64.7%	60.9%	
**Changes in Intimacy and Sexual Interest**			
Mean score	5.8 (1.4)	5.8 (0.9)	
< 6	29.4%	26.1%	>0.9
≥ 6	70.6%	73.9%	

^a^Includes one participant who had received no treatment at the time of sampling

Survivors with endometrioid histology (which is more common and has a more favorable prognosis) had significantly lower alpha (Shannon p = 0.005, Chao1 p = 0.009) and beta (Bray-Curtis p = 0.005) diversity (**Fig 2**) and were more likely to have *Lactobacillus*-dominant vagitypes (85.7% vs. 63.6%, p = 0.05, **Table 2**) than those with non-endometrioid histology.

**Fig 5:**
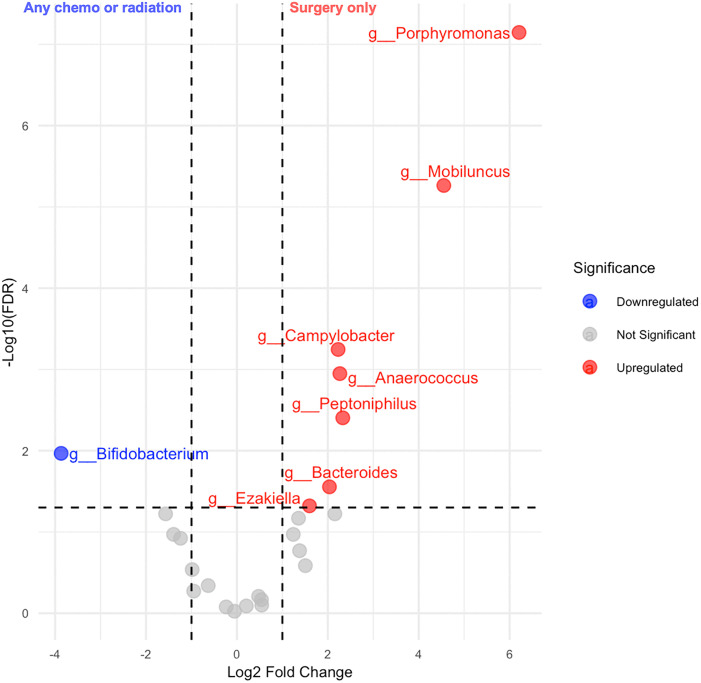
Volcano plot of differentially abundant bacterial genera in vaginal microbiome according to cancer treatment (any chemotherapy/radiation vs. surgery only).

Women who received surgery alone had less diverse vaginal communities (potentially indicating a more favorable vaginal microbiome) than those who received chemotherapy or radiation (Shannon p = 0.04, Bray-Curtis p = 0.004) however, the Chao1 index was not statistically significant (p = 0.1). **Fig 3** shows the alpha and beta diversity patterns of vaginal samples according to cancer treatment status. Participants with chemotherapy or radiation were less likely to have a *Lactobacillus*-dominant vagitype (23% vs. 77%, p = 0.006). Using univariate analysis, we identified discriminative genera in the vaginal microbiome according to cancer treatment status (**Fig 5**). Among eight discriminative genera by treatment type, most genera (7/8) were more abundant in vaginal microbiome of participants that received chemotherapy or radiation.

Distributions by race, histology, and treatment status are provided in **S2 Table**. In exploratory analyses, we restricted analyses to survivors with the more common endometrioid histology (N = 29) to assess whether cancer treatment associations persisted independently of histology. Among endometrioid histology samples, we did not observe statistically significant differences in alpha (Shannon p = 0.6, Chao1 p = 0.9) or beta (Bray-Curtis p = 0.08) diversity according to treatment status. However, among women with *Lactobacillus*-dominant vagitypes, most received surgery only (80%) rather than radiation or chemotherapy (20%). Among those with a non-*Lactobacillus*-dominant vagitype, radiation/ chemotherapy was more common (54%) than surgery only (46%); although this difference in proportions was not statistically significant (p = 0.1, **S3 Table**).

### Vaginal microbial composition and patient-reported sexual function

The distribution of SVQ scores by vagitype is shown in **Table 2**. Sexual interest (p = 0.8), global sexual satisfaction (p = 0.2), intimacy (p = 0.3), and changes in intimacy and sexual interest (p > 0.9) did not significantly vary between the *Lactobacillus*-dominant and non-dominant groups (**Table 2**). No significant differences in alpha or beta diversity patterns were observed for SVQ global sexual satisfaction (Shannon p = 0.6, Chao1 p = 0.6, Bray-Curtis p = 0.9), intimacy (Shannon p = 0.4, Chao1 p = 0.4, Bray-Curtis p = 0.3), and changes in interest and sexual interest (Shannon p = 0.3, Chao1 p = 1.0, Bray-Curtis p = 0.5) scores above versus below the median.

## Discussion

Our study assessed the feasibility of self-collection of stool samples and vaginal swabs for microbiome profiling among 50 endometrial cancer survivors living in North Carolina. The vast majority of participants successfully completed sample collection, demonstrating feasibility. Participants indicated a higher degree of comfort with the vaginal swab collection process compared to stool collection. In our pilot sample, stool and vaginal microbial diversity did not statistically significantly vary by race, although *Lactobacillus*-dominant vagitypes appeared less common among Black participants compared to White. Participants who underwent surgical treatment only appeared to have less diverse vaginal microbial communities, potentially indicating better vaginal health. Stool microbial diversity did not appear to be related to cancer treatment status more than a year after diagnosis. Stool and vaginal microbial composition were not strongly correlated with patient-reported bowel or sexual function, respectively.

Our findings reinforce the feasibility and acceptability of vaginal self-sampling to investigate vaginal microbiome health among gynecologic cancer survivors. Similar to our results, a systematic review of vaginal self-sampling for diagnosis of sexually transmitted infections reported an overwhelming majority of participants (86%) expressed a willingness to use self-sampling in the future, and preferred sampling at home (65%) over clinic-based sampling (44%) [60].

In our analysis, stool and vaginal microbiome profiles were not statistically different by race. There are limited prior studies of potential racial variation in stool or vaginal microbiome composition specific to gynecologic cancer survivors. A study of 42 cervical cancer survivors found no significant differences in alpha or beta diversity in stool microbiome across racial groups [61]. Similar to our results, no differences in diversity were reported in vaginal samples from 34 black and 29 white endometrial and cervical cancer survivors [62,63]. However, in a much larger U.S. study of women without cancer, African American women (n = 960) had significantly higher vaginal alpha diversity than women of European ancestry (n = 330); beta diversity did not vary between groups [40]. For the gut microbiome, a study collecting stool samples from 1,673 healthy adults in the United States found that African American participants had significantly lower alpha diversity compared to White individuals [45]. Future studies with larger and more diverse cohorts of endometrial cancer survivors are needed to better understand potential racial variation in vaginal microbiome composition.

In our analysis, vaginal microbiome profiles of endometrial cancer survivors varied according to cancer treatment status with less favorable (more diverse) profiles associated with chemotherapy or radiation compared to surgery alone. In previous studies, higher alpha diversity of the vaginal microbiome has been associated with dysbiosis [26,64,65] and negative health outcomes such as vaginosis [66,67], cervical lesions, cervical cancer [68], and preterm birth [69]. Radiation or chemotherapy may impair vaginal health by adversely influencing the microbiome equilibrium. Alternatively, these differences could reflect sustained differences from pre-diagnosis profiles, whereby more diverse vaginal microbiome profiles are related to worse prognosis diagnoses which call for more intensive therapies. In our analysis, both non-endometrioid histology and intensive cancer treatment were associated with higher vaginal microbiome diversity. Non-endometrioid disease is also more likely to be treated with more intensive cancer therapies. Our analysis had limited ability to evaluate cancer treatment associations in analyses stratified by histology due to sample size. Cancer treatment associations were not statistically significant when restricted to the more common endometrioid histology; however, the majority of endometrioid histologies treated with surgery had a *Lactobacillus*-dominant vagitype; while the majority of those treated with radiation or chemotherapy had non-*Lactobacillus* dominant vagitypes.

To our knowledge, two prior studies have assessed endometrial cancer histology in relation to vaginal microbiome diversity. The first compared vaginal microbiome composition between 20 women with high-grade (non-endometroid) and 30 women with low-grade endometrial cancer and 10 women with benign endometrial conditions treated at the University of Miami Hospital, Sylvester Comprehensive Cancer Center or Jackson Memorial Hospital in Florida. Vaginal microbial communities were significantly more diverse among women with high grade (p = 0.02) and serous (the most common non-endometroid histology, p = 0.07) histologies relative to benign conditions; however, differences were not statistically significant between high and low grade endometrial cancers [70]. At the Albert Einstein College of Medicine in New York, investigators compared cervicovaginal swabs collected at hysterectomy from 35 women—10 with benign conditions and 25 with uterine cancer (14 endometroid and 11 serous tumors). The Shannon index showed higher vaginal microbiome diversity for women with endometrioid and serous tumors compared to benign conditions; however, this difference was not statistically significant (Kruskal-Wallis p = 0.3) [71]. These findings generally align with our observation that survivors with endometrioid histologies had lower, and potentially more favorable, vaginal microbiome diversity than those with non-endometrioid histologies.

*Lactobacillus* species have been well-studied for their important role in maintaining vaginal health by producing lactic acid, creating an acidic environment and thus impairing the colonization of pathogenic bacteria and reducing inflammation [72–74]. Restoring *Lactobacillus*-dominant vaginal microbiome profiles after cancer therapy could be beneficial for patients to reduce the risks of infection and inflammation. *Lactobacillus* therapies, such as vaginal tablets, intravaginal administration of probiotics [72,75,76], and targeted probiotic or dietary strategies have potential to restore a *Lactobacillus*-dominant microbiome profile. Such post-treatment approaches could offer a complementary strategy to manage genitourinary or gastrointestinal side effects of intensive cancer treatment to improve health outcomes and quality of life of endometrial cancer survivors [72,75,76].

We identified eight statistically significantly distinguished genera between surgery only and chemotherapy/radiation cancer treatment subgroups. These included *Anaerococcus* [77], *Porphyromonas* [78,79], *Mobiluncus* [80], which have been associated with bacterial vaginosis and dysbiosis in the vaginal microbiome. The increased abundance in treated individuals may reflect impairment of vaginal microbiome health. In contrast, participants with less intensive treatment had higher levels of *Bifidobacterium*, a genus typically considered protective for maintaining vaginal microbiome homeostasis by producing acetic acid [81,82], was more abundant in the group that received surgery only. A dysbiotic vaginal microbiome profile after chemotherapy or radiation may increase the risk of inflammation and thus impact quality of life or recurrence risk of cancer survivors; the vagina is the most common site for endometrial cancer recurrence [83,84].

Results from our study should be considered in the context of its limitations and strengths. Stool and vaginal swab samples were collected at a single time point approximately 16 months after diagnosis. We did not have access to repeated samples to compare microbiome profiles within individuals before and after cancer treatment, or to compare to a control group of healthy women. Without these comparisons, we cannot definitively attribute the microbiome alterations we observed to the presence of endometrial cancer itself, its treatment modalities, or to other unmeasured exposures. To fill this gap, future study designs should consider inclusion of women without endometrial cancer or longitudinal sample collection to enable more direct identification of specific disease‑ and treatment-related changes in microbial communities.

The small sample size of our pilot did not allow us to compare detailed treatment categories or adjust for other demographic or cancer-related characteristics in our analyses. Future studies with larger sample sizes are needed to address the impact of specific chemo- or immunotherapies and radiation modalities on vaginal and gut microbiome composition in endometrial cancer survivors. Dietary intake data were not collected in CECS cohort and thus could not be included as a covariate in our models but should be considered in future work given the influence of diet on gut microbiome composition. Finally, the bacterial identification methods lacked the resolution to distinguish between species, limiting our ability to analyze differences between specific *Lactobacillus* species in vaginal samples. In future work, approaches such as shotgun metagenomic sequencing can be used to provide strain level resolution. As a pilot study, our sample was small by design (n = 50) with limited statistical power and generalizability. However, this work provides a valuable foundation for the design of future studies to validate and extend our findings to enhance clinical care for endometrial cancer survivors. Future studies should incorporate functional and mechanistic approaches to validate causal relationships and identify the biological roles of key taxa in endometrial cancer survivorship and treatment response.

Our initial pilot study provided an opportunity to demonstrate the feasibility and acceptability of self-collection of stool and vaginal swab samples for microbiome profiling among endometrial cancer survivors. These specimens are stable and non-hazardous for mail-based collection [60,85–87], increasing research opportunities for large sample collection in epidemiologic cohorts, such as the Carolina Endometrial Cancer Study. With future research, microbiome profiling has potential to inform personalized survivorship care to gauge treatment response, manage treatment-related symptoms, and predict recurrence.

## Supporting information

S1 Fig Alpha and Beta diversity of stool microbiome samples according to CRADI-8 score tertiles: a. Alpha diversity (Shannon index); b. Beta diversity (Bray-Curtis).
(DOCX)

S2 Table Distributions of tumor histology and cancer treatment status according to race among participants with treatment information (N = 39). (DOCX)

S3 Table Distributions of cancer treatment status and vagitype among participants with endometroid carcinoma (N = 29). (DOCX)
