# Supplementary material for: Stool and vaginal microbiome profiles patterns among Black and White endometrial cancer survivors: A pilot study in North Carolina
Source: PLoS One. 2026 Jan 23;21(1):e0336772. doi: 10.1371/journal.pone.0336772 (PMC12829856; doi:10.1371/journal.pone.0336772)
Supplement: S1 Fig — Alpha diversity (Shannon index); b. Beta diversity (Bray-Curtis). (DOCX) [file pone.0336772.s001.docx]

**S1 Figure:** Alpha and Beta diversity of stool microbiome samples according to CRADI-8 score tertiles: a. Alpha diversity (Shannon index); b. Beta diversity (Bray-Curtis)

**
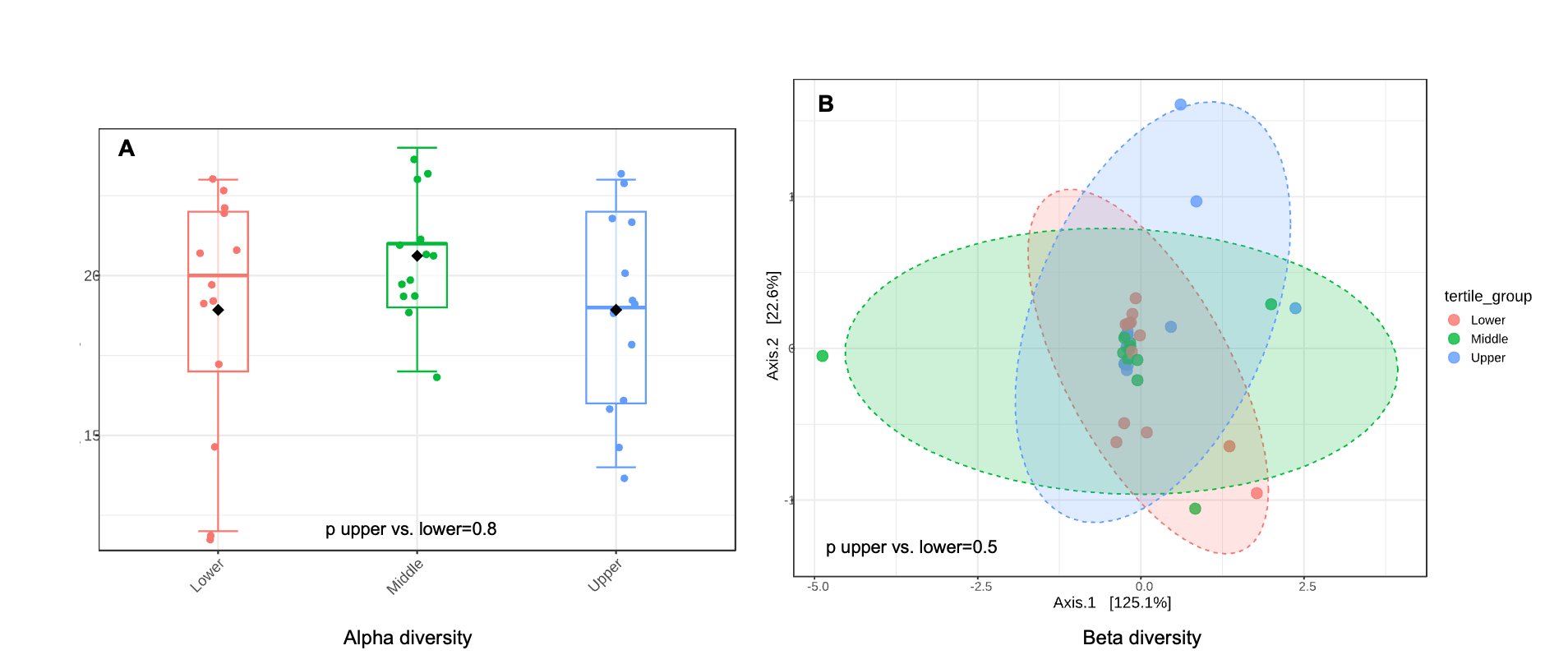
**
