# Supplementary material for: Stool and vaginal microbiome profiles patterns among Black and White endometrial cancer survivors: A pilot study in North Carolina
Source: PLoS One. 2026 Jan 23;21(1):e0336772. doi: 10.1371/journal.pone.0336772 (PMC12829856; doi:10.1371/journal.pone.0336772)
Supplement: S2 Table — (DOCX) [file pone.0336772.s002.docx]

| **S2 Table:** Distributions of tumor histology and cancer treatment status according to race among participants with treatment information (N=39) | | |
| --- | --- | --- |
| **Tumor histology** | **Surgery only^a^** | **Any radiation or chemotherapy** |
| Black participants (N=19) |  |  |
| Endometrioid | 88.9% | 40.0% |
| Non-endometrioid | 11.1% | 60.0% |
|  |  |  |
| White participants (N=20) |  |  |
| Endometrioid | 100.0% | 60.0% |
| Non-endometrioid | 0.0% | 40.0% |
| ^a^ Includes one participant who had received no treatment at the time of sampling | | |
