# Supplementary material for: Stool and vaginal microbiome profiles patterns among Black and White endometrial cancer survivors: A pilot study in North Carolina
Source: PLoS One. 2026 Jan 23;21(1):e0336772. doi: 10.1371/journal.pone.0336772 (PMC12829856; doi:10.1371/journal.pone.0336772)
Supplement: S3 Table — (DOCX) [file pone.0336772.s003.docx]

| **S3 Table:** Distributions of cancer treatment status and vagitype among participants with endometroid carcinoma (N=29) | | |
| --- | --- | --- |
| **Cancer Treatment** | ***Lactobacillus*-dominant**  N=16 | **Non-*Lactobacillus*-dominant**  N=13 |
| Surgery only^a^ | 80.0% | 46.2% |
| Any radiation or chemotherapy | 20.0% | 53.8% |
| ^a^ Includes one participant who had received no treatment at the time of sampling | | |
